# Diagnostic and Prognostic Implications of a Serum miRNA Panel in Oesophageal Squamous Cell Carcinoma

**DOI:** 10.1371/journal.pone.0092292

**Published:** 2014-03-20

**Authors:** Caiyun Wu, Cheng Wang, Xiaocui Guan, Yuxiu Liu, Demin Li, Xiaojun Zhou, Yanni Zhang, Xi Chen, Junjun Wang, Ke Zen, Chen-Yu Zhang, Chunni Zhang

**Affiliations:** 1 Department of Clinical Laboratory, Jinling Hospital, School of Life Sciences, Nanjing University, Nanjing, China; 2 Jiangsu Engineering Research Center for microRNA Biology and Biotechnology, State Key Laboratory of Pharmaceutical Biotechnology, School of Life Sciences, Nanjing University, Nanjing, China; 3 Department of Scientific Research Management, Jinling Hospital, Clinical School of Medical College, Nanjing University, Nanjing, China; 4 Department of Cardio-thoracic Surgery, Jinling Hospital, Clinical School of Medical College, Nanjing University, Nanjing, China; 5 Department of Pathology, Jinling Hospital, Clinical School of Medical College, Nanjing University, Nanjing, China; 6 Department of Medical Oncology, Cancer Hospital of Xuzhou, Xuzhou, China; Memorial Sloan-Kettering Cancer Center, United States of America

## Abstract

**Background and Aim:**

Circulating microRNAs (miRNAs) are potential biomarkers for cancer detection; however, little is known about their prognostic impact on oesophageal squamous cell carcinoma (ESCC). The current study aims to uncover novel miRNAs for prognostic biomarkers in ESCC patients.

**Patients and Methods:**

We initially screened the expression of 754 serum miRNAs using TaqMan Low Density Array in two pooled samples respectively from 28 ESCC and 28 normal controls. Markedly upregulated miRNAs in ESCC and some miRNAs reported to be differently expressed in ESCC tissue were then validated individually by RT-qPCR in another 83 patients and 83 controls arranged in two phases. The changes of the selected miRNAs during the esophagectomy and their prognostic value were examined.

**Results:**

Seven serum miRNAs were found to be significantly higher in ESCC than in controls; namely, miR-25, miR-100, miR-193-3p, miR-194, miR-223, miR-337-5p and miR-483-5p (*P*<0.0001), and the area under the receiver-operating-characteristic (ROC) curve (AUC) for the seven-miRNA panel was 0.83 (95% CI 0.75–0.90). Most of these miRNAs declined markedly in postoperative samples versus preoperative samples (*P*<0.05). Moreover, high level of miR-25 was significantly correlated with shorter overall survival in patients (*P* = 0.027). Cox regression analysis identified lymph node metastasis, miR-25 and miR-100 as the independent risk factors for overall survival (hazard ratio (HR) 2.98 [1.36–6.55], *P* = 0.006; HR 3.84 [1.02–14.41], *P* = 0.029; HR 4.18 [1.21–14.50], *P* = 0.024, respectively).

**Conclusion:**

The seven serum miRNAs could potentially serve as novel biomarkers for ESCC; moreover, specific miRNAs such as miR-25 and miR-100 can predict poor survival in ESCC.

## Introduction

Oesophageal carcinoma is regarded as the eighth most common malignant cancer and the sixth most common cause of death from cancer, with 482,000 new cases and 406,000 deaths (5.4% of all cancer deaths) in 2008 [1], [2]. Oesophageal squamous cell carcinoma (ESCC) is the most common pathological type in Asian countries (∼90%), and in China ESCC is the second most common cancer and the fourth-leading cause of cancer-related death [2], [3]. Although surgical techniques and perioperative management have progressed, ESCC remains one of the most aggressive carcinomas of the gastrointestinal tract and has a 5-year survival rate of approximately 20–40% even after curative surgery [2]–[5]. The poor prognosis of ESCC patients results from diagnosis at a late stage and the poor efficacy of treatment. Blood-based tests appear to be particularly attractive as they are minimally invasive. However, to date, no blood test is currently available. Therefore, the development of new methods for the detection, prognosis and monitoring of ESCC are urgently needed to reduce disease morbidity and mortality.

MicroRNAs (miRNAs) are small noncoding RNAs 19–24 nucleotides in length that are thought to be involved in the development of cancer. Recent studies by our group and others have demonstrated that miRNAs are stably detectable in the blood and can serve as useful biomarkers for cancer [6]–[10]. Unlike in some other cancers, the role of circulating miRNAs as biomarkers for detection and prognosis of ESCC has not been thoroughly studied [11]. In a recent study, we identified a seven-miRNA profile that was significantly increased in serum from patients with ESCC compared with normal individuals; these miRNAs could be used as biomarkers for ESCC [7]. In this previous study, we evaluated the potential of these miRNAs as markers for early detection only, not for prognosis or treatment response [7]. Subsequently, a few other dysregulated serum miRNAs have been identified by other groups [5], [12]–[16]. With a very poor 5-year survival rate in ESCC, markers to assess prognosis or treatment effect are equally important. However, to date, there is still a lack of extensive analysis of the dynamic changes and prognostic value of serum miRNAs in ESCC patients [11].

Therefore, in the present study, we employed the reverse transcription (RT)-PCR-based TaqMan Low Density Array scanning combined with a literature review followed by confirmation with a quantitative reverse-transcription PCR (RT-qPCR) assay based on an reliable internal reference gene to uncover novel serum miRNAs that could be of potential use in monitoring tumor dynamics and predicting the prognosis of ESCC patients.

## Methods

### Study design and subjects

The present study enrolled 111 ESCC, 15 gastric cancer and 20 hepatocarcinoma patients, all of whom were newly diagnosed and were treated at Jinling Hospital (Nanjing, China) and the Cancer Hospital of Xuzhou (Xuzhou, China) between 2011 and 2014. All the patients underwent a tumorectomy before any adjunctive therapy. Pathology specimens from all patients enrolled in the study were centrally reviewed using the current WHO classification scheme [17]. Patients with acute infections or other types of cancer were excluded from our study. Of the ESCC patients, 63 were followed up until death or until July 31, 2013. The median follow-up time was 19.6 months (mean± SD: 19.1 ± 3.7 months). In addition, 111 individuals who were recruited from a large pool of individuals seeking a routine health checkup at Jinling Hospital and showed no evidence of disease were selected as non-cancer controls.

Written informed consent was obtained from all patients and healthy participants prior to the study. The study protocol was approved by the ethics committees of Jinling Hospital and Cancer Hospital of Xuzhou. The study was performed in accordance with the Declaration of 1975 Helsinki and the REMARK guidelines for biomarker studies.

### Blood sampling

The pre-operative serum samples of ESCC patients were collected 1–7 days (median  = 3 days; mean ± SD: 3.7±1.7 days) before surgery and the post-operative samples were obtained 7–10 days post-surgery. A total of 3 mL venous blood was collected from each study participant after 12 h of overnight fasting. Each blood sample was immediately centrifuged at 3000 g for 5 min at room temperature, followed by a 5 min high-speed centrifugation step of the supernatant at 10,000 g at 4°C. The samples were stored at −80°C until analysis. The storage time of serum samples was ranging from 5 days to 334 days (mean ± SD: 213±122 days).

### RNA isolation

For the TaqMan Low Density Array, equal volumes of sera from 28 ESCC patients and 28 controls (500 μL each) were pooled separately to form the case and control sample pools (each pool contained 14 mL), respectively. TRIzol reagent (Invitrogen, Carlsbad, CA) was used to extract total RNA from each pool of serum samples as previously described [6]. The aqueous phase was subjected to 3 steps of acid phenol/chloroform purification to eliminate protein residues before isopropyl alcohol precipitation. The resulting RNA pellet was dissolved in 20 μL ribonuclease-free water and stored at −80°C until further analysis. The RNA was quantified by using a NanoDrop 2000 UV-Vis Spectrophotometer (Thermo Fisher Scientific Inc.). The amounts of RNA in the two yields were 7.4 μg and 6.4 μg, respectively.

RNA from the serum samples in each cohort was prepared for individual RT-qPCR assay at roughly the same time. Total RNA was extracted from 100 μL serum with a 1-step phenol/chloroform purification protocol as previously described [9]. Briefly, an amount of 100 μL of serum was mixed with 200 μL acid phenol, 200 μL chloroform, and 300 μL RNase-free water. The mixture was vortex-mixed vigorously and centrifuged at room temperature for 15 min. After phase separation, the aqueous layer was mixed with 1.5 volumes of isopropyl alcohol and 0.1 volumes of 3 mol/L sodium acetate (pH 5.3). This solution was stored at −20°C for 1 h. The RNA pellet was collected by centrifugation at 16,000 g for 20 min at 4°C. The resulting RNA pellet was washed once with 750 mL/L ethanol and dried for 10 min at room temperature. Typically, after extracting total RNA from 100 μL serum, the amounts of RNA in the yields were in the range of approximate 50–100 ng. Finally, the pellet was dissolved in 20 μL of RNase-free water and stored at −80°C until further analysis.

### Serum miRNA assay using TaqMan Low Density Array

For the TaqMan Low Density Array, reverse transcription was performed using the TaqMan MicroRNA Reverse Transcription Kit and Megaplex RT Primers as previously described [18]. Briefly, 3 μL total RNA were added to 4.5 μL of the RT reaction mix (Megaplex RT Primers 10X, dNTPs with dTTP 100 mmol, MultiScribe Reverse Transcriptase 50 U/μL, 10× RT Buffer, MgCl_2_ 25 mmol, RNase Inhibitor 20 U/μL and nuclease-free water). After incubation on ice for 5 min reverse transcription was performed using a thermal cycler (UNO-Thermoblock, Biometra, Göttingen, Germany). MicroRNA profiling of 754 different human microRNAs was then performed using the TaqMan Low Density Array (TaqMan Array Human MicroRNA A+B Cards Set v3.0) (Life Technologies, Carlsbad, CA). In order to increase the sensitivity of the TaqMan Low Density Array, a pre-amplification was performed after the reverse transcription. All steps were performed using a 7900 HT Fast Real-Time PCR System (Applied Biosystems, Foster City, CA) and all reactions were performed as specified in the protocols of the manufacturer. miRNA concentrations were presented as threshold cycle (Cq) values and normalized to an internal control recommended by the manufacturer on the calculated Cq of each miRNA (ΔCq). The fold changes of miRNA expression were calculated using the equation 2^−ΔΔCq^.

### Individual RT-qPCR assays of serum miRNAs

A TaqMan probe–based RT-qPCR assay was performed according to the manufacturer's instructions (7300 Sequence Detection System; Applied Biosystems), with a minor modification as described previously [18]. Briefly, the reverse transcription reaction was carried out in 10 μL containing 2 μL of extract RNA, 1 μL of 10 mmol/L dNTPs, 0.5 μL of AMV reverse transcriptase (TaKaRa), 1 μL of a stem-loop RT primer (Applied Biosystems), 2 μL of 5 X reverse transcription buffer and 3.5 μL of diethylpyrocarbonate (DEPC) water. For synthesis of cDNA, the reaction mixtures were incubated at 16°C for 30 min, at 42°C for 30 min, at 85°C for 5 min, and then held at 4°C. Real-time PCR was performed (1 cycle of 95°C for 5 min, and 40 cycles of 95°C for 15 sec and 60°C for 1 min) with an Applied Biosystems 7300 Sequence Detection System. The reaction was performed with a final volume of 20 μL containing 1 μL of cDNA, 0.3 μL of Taq, 0.33 μL of hydrolysis probe (Applied Biosystems), 1.2 μL of 25 mmol/L MgCl_2_, 0.4 μL of 10 mmol/L dNTPs, 2 μL of 10 X PCR buffer, and 14.77 μL of DEPC water. The product/catalog numbers of miRNAs for the Applied Biosystems miRNA RT-PCR assays were shown in Table S1. All reactions, including no-template controls, were performed in triplicate. A combination of let-7d, let-7g and let-7i (let-7d/g/i), which shows low variability between ESCC and normal controls and is statistically superior to the most commonly used reference genes in the quantification of serum miRNAs was measured as endogenous control for normalizing the data of experimental RT-qPCR (Figure S1) [19]. The total amount of let-7d/g/i trio was simultaneously measured in a same RT-qPCR reaction [19]. In brief, let-7d, let-7g and let-7i in 2 μL of total RNA were reverse-transcribed in a single reaction using specific RT Primer pool, a mixture of stem-loop primers of let-7d, let-7g and let-7i (in the ratio of 1∶1∶1). Accordingly, real-time PCR was performed using TaqMan miRNA probe pool of let-7d, let-7g and let-7i (in the ratio of 1∶1∶1). Then relative levels of miRNAs were normalized to a let-7d/g/i and were calculated using the 2^−ΔΔCq^ method. ΔCq was calculated by subtracting the Cq values of let-7d/g/i from the average Cq values of the target miRNAs. ΔΔCq was then determined by subtracting the ΔCq of the normal controls from the ΔCq of the cases.

### Serum carcinoembryonic antigen determination

The serum values of carcinoembryonic antigen (CEA) were measured using a commercial kit (CEA fluoroimmunometric assay, Beckman Coulter Inc., Fullerton, CA) on the Beckman Coulter UniCel™ DxI 800 Access® Immunoassay System.

### Data analysis

All the statistical analyses were performed using the Statistical Analysis System software SPSS 16.0. Data were presented as the mean ± SEM for miRNAs or mean ± SD for other variables. A Student's *t*-test or two-sided χ^2^ test was used to compare differences in variables between two groups. A *P*-value <0.05 was considered statistically significant. For miRNAs, we constructed receiver-operating-characteristic (ROC) curves and calculated the area under the ROC curves (AUC) to evaluate the predictive power of candidate miRNA for ESCC. Risk score analysis was performed to evaluate the associations between the concentrations of the serum miRNAs and ESCC as previously described (deposited in the Supplementary data) [9]. The risk score of each miRNA, denoted as s, was set to 1 if the expression level was greater than the upper 95% reference interval for the corresponding miRNA level in controls and to 0 otherwise. A risk score function (RSF) to predict ESCC was defined according to a linear combination of the expression level for each miRNA. For example, the RSF for sample i using information from seven miRNAs was: rsf_i_ = ∑^7^_j-1_W_j_.s_ij_. In the above equation, s_ij_ is the risk score for miRNA j on sample i, and W_j_ is the weight of the risk score of miRNA j. To determine the Ws, seven univariate logistic regression models were fitted using the disease status with each of the risk scores. The regression coefficient of each risk score was used as the weight to indicate the contribution of each miRNA to the RSF. Samples were ranked according to their RSF and then divided into a high-risk group, representing the predicted ESCC cases, and a low-risk group, representing the predicted control individuals. Frequency tables and ROC curves were then used to evaluate the diagnostic effects of the profiling and to find the appropriate cutoff point. In addition, we utilized the Kaplan-Meier method using death as an outcome to determine the associations between overall survival (OS) and serum miRNA levels. The OS was defined as the interval between surgery and death or the last follow-up examination. Statistical significance was calculated using the log-rank test. Stepwise univariable and multivariable Cox regression was performed to analyze factors related with OS.

## Results

### Expression profile of miRNAs in the serum of ESCC patients

**Figure 1:**
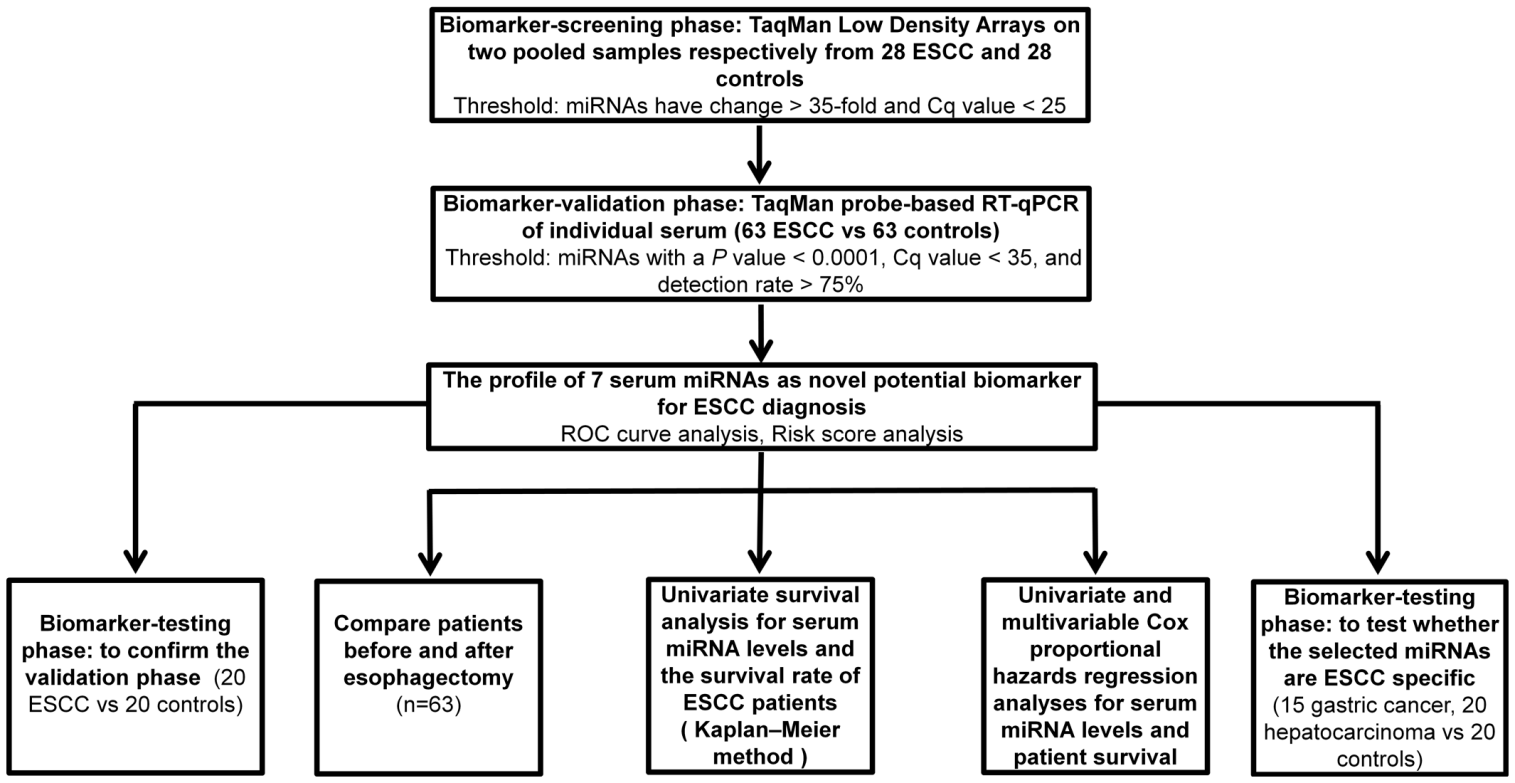
A flow chart of the experimental design.

A multiphase case control study was designed to identify novel serum miRNAs as surrogate biomarkers for diagnosing, monitoring tumor dynamics and predicting the prognosis of ESCC patients (Figure 1). To screen miRNAs that are upregulated in ESCC, we comparatively performed and analyzed the global miRNA expression profiles in two pooled serum samples from patients with ESCC and normal controls by TaqMan Low Density Array. The 28 controls were exactly matched with the 28 ESCC patients in age, sex, smoking status and alcohol consumption (Table S2). Of the 754 miRNAs scanned, twenty six were upregulated in ESCC (>2-fold increase) (Table S3). We selected miRNAs that satisfied two criteria for additional RT-qPCR validation: Cq values were <25 in the ESCC sample and showed at least a 35-fold increase in the ESCC sample compared to the control sample. As a result, six miRNAs including miR-198, miR-337-5p, miR-216a, miR-1247, miR-193a-3p and miR-194 were identified and subjected to further analyses (Table S3).

### Validation of candidate miRNAs by RT-qPCR

**Figure 2:**
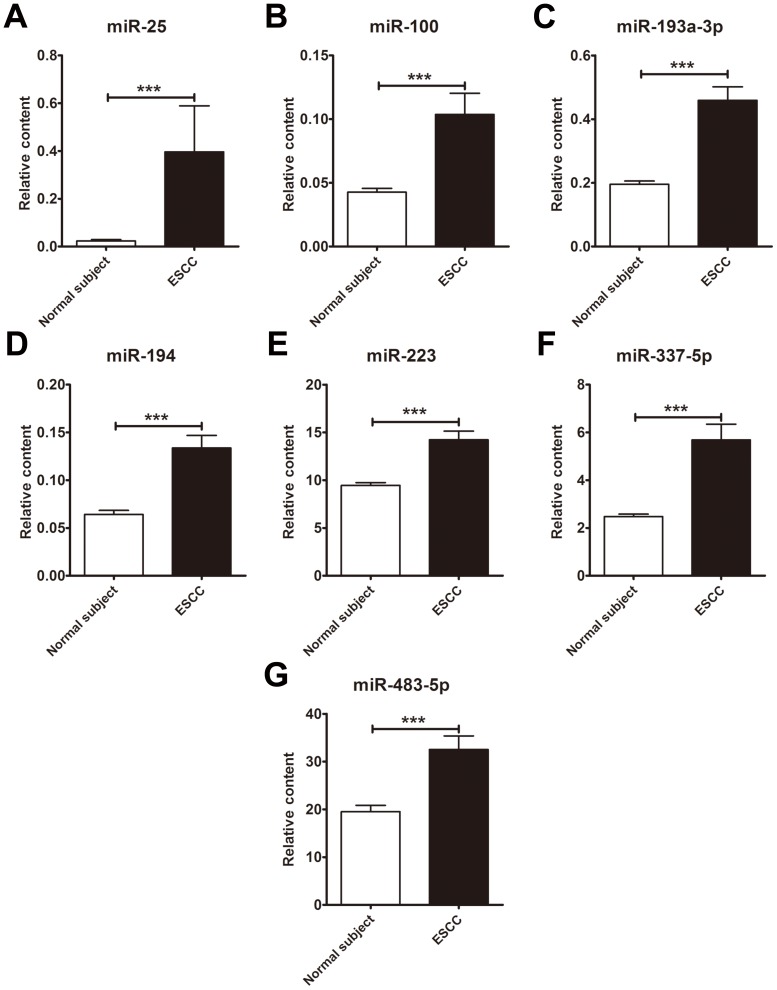
The significantly elevated miRNAs in sera from patients with oesophageal squamous cell carcinoma (ESCC). (A–G) The contents of seven miRNAs including miR-25, miR-100, miR-193-3p, miR-194, miR-223, miR-337-5p and miR-483-5p were validated in sera from ESCC patients (n = 63) and normal subjects (n = 63) by RT-qPCR assay. The relative levels of the seven miRNAs were normalized to let-7d/g/i and calculated using the 2^−ΔΔCq^ method. Error bars  =  SEM. Asterisks refer to *P*<0.0001.

Although the TaqMan Low Density Array is a RT-PCR-based assay, it was performed on only one pooled sample for each of ESCC and control cases, and might yield some inaccurate information due to individual variation. The array results must be validated using RT-qPCR assay performed at an individual level of serum samples. Therefore, the six markedly upregulated miRNAs selected from the Low Density Array were further examined in an independent cohort of 63 ESCC patients by an individual RT-qPCR assay. As summarized in Table 1 and Table S4, there were no significant differences between the ESCC patients and the control individuals based on age distribution, sex, smoking status, alcohol consumption and other diseases. As a result, all the six serum miRNAs were found to be increased in ESCC patients as compared with normal controls in RT-qPCR assay, and four of them (including miR-337-5p, miR-1247, miR-193a-3p and miR-194) displayed statistically significantly increased (compared with controls, at least *P*<0.0002) (Table S5). In addition, five miRNAs (miR-7, miR-25, miR-100, miR-483-5p and miR-223), which have previously been reported to be dysregulated in ESCC tissues, were also included in the confirmation analysis. The relative amounts of all the five miRNAs were found to be significantly increased in ESCC patient serum as compared with that from normal individuals (*P*-values ranging from <0.05 to <0.0001)(Table S5). Of the markedly increased serum miRNAs examined in RT-qPCR, seven with *P*-values <0.0001 including miR-25, miR-100, miR-193a-3p, miR-194, miR-223, miR-337-5p, miR-483-5p were selected as candidates for further analyses (Figures 2A–2G).

**Table 1:** Demographic and clinical features of the oesophageal squamous cell carcinoma (ESCC) patients and normal controls whose sera were examined with individual RT-qPCR assays in the validation cohort.^1^

Variables	ESCC (n = 63)		Normal controls (n = 63)		*P* - value
	No.	%	No.	%	
**Average age (years)**	62.0±8.3		59.7±6.8		0.089^2^
**Age (years)**					0.470^3^
≤59	24	38.1	29	46.0	
>59	39	61.9	34	54.0	
**Sex**					1.000^3^
Male	55	87.3	55	87.3	
Female	8	12.7	8	12.7	
**Smoking status**					0.373^3^
Ever and Current	36	57.1	30	47.6	
Never	27	42.9	33	52.4	
**Alcohol consumption**					0.476^3^
Ever or Current	33	52.4	28	44.4	
Never	30	47.6	35	55.6	
**Differentiation grade**					
High	5	7.9			
High -Middle	7	11.1			
Middle	28	44.4			
Middle-Low	7	11.1			
Low	16	25.4			
**TNM Stage**					
I	12	19.0			
II	39	61.9			
III	10	15.9			
IV	2	3.1			
Esophagectomy	63	100			
**Chemoradiation after surgery**					
Yes	58	92			
No	5	8			
**Family history of cancer**					
Family history of ESCC	1	1.6			
No family history of ESCC	62	98.4			
**Significant cardiac dysfunction^4^**					0.243^3^
Yes	0	0	3	5	
No	63	100	60	95	
**Neurologic disease or diabetes**					0.476^3^
Yes	2	3.2	0		
No	61	96.8	63		

1 Data are mean ± standard deviation. ^2^ *P*, student-*t* test;^3^ *P*, two-sided χ^2^ test; ^4^ Significant cardiac dysfunction mainly refers to arrhythmia, heart failure, myocardial infarction or myocarditis.

The selected seven miRNAs were further examined by RT-qPCR in an additional validation cohort (testing phase) consisting of 20 ESCC patients and 20 matched controls. Consistent with the results from the former cohort, the serum contents of the seven miRNAs were significantly higher in the cancer cases than in the control individuals (at least *P*<0.05) (Table S6).

### Diagnostic accuracy of the selected serum miRNAs in ESCC

**Figure 3:**
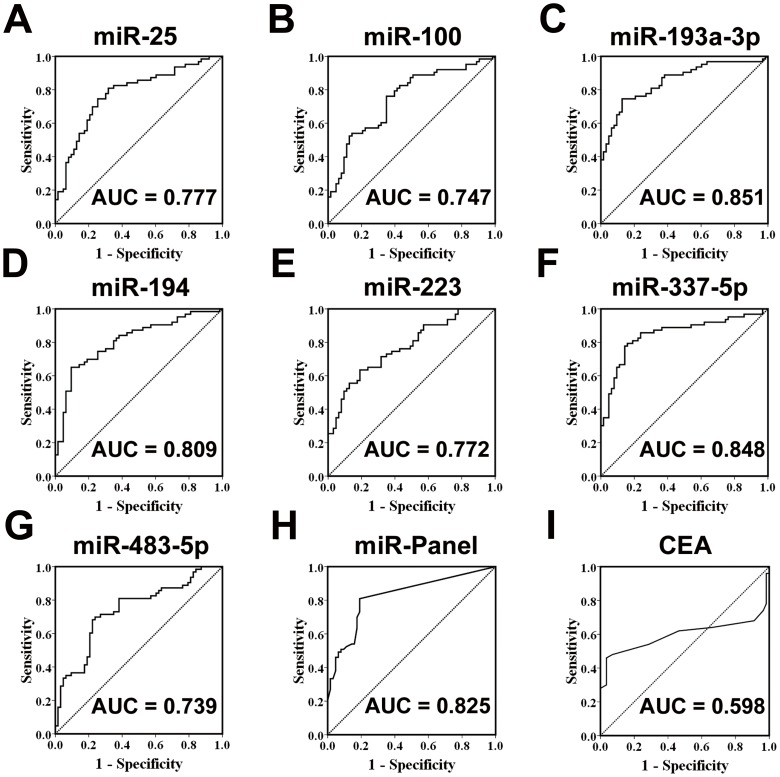
ROC curves to compare the ability of miRNA to distinguish oesophageal squamous cell carcinoma (ESCC) from the normal controls. (A) miR-25, (B) miR-100, (C) miR-193-3p, (D) miR-194, (E) miR-223, (F) miR-337-5p, (G) miR-483-5p, (H) the seven-miRNA panel and (I) carcinoembryonic antigen (CEA).

Subsequently, to assess the diagnostic usefulness of the selected seven serum miRNAs in discriminating between ESCC and normal controls, an analysis of ROC was conducted in the validation cohort and testing cohort, respectively. The AUCs for these miRNAs ranged from 0.74 to 0.85 for the serum samples in the validation cohort of 63 patients and 63 controls (Figures. 3A–3G; Table S7). Furthermore, the AUC for the combination of the seven miRNAs was 0.83 (95% CI 0.75–0.90) for ESCC (Figure 3H; Table S7). With an optimal cutoff value (0.764), at which the sum of the sensitivity and specificity was maximal, the specificity and sensitivity for ESCC was 81% (Table 2). For the serum samples in the testing cohort, the AUC for the seven miRNA panel was 0.93 (95% CI 0.86–1.01) (Table S8). In addition, we compared the AUC of seven-miRNA panel with that of CEA, and found the AUC of the panel was higher than that of CEA (0.60, 95% CI 0.47–0.72) (Figure 3I;Table S7). Together, these results indicate that the identified miRNAs alone or in combination can discriminate between ESCC cases and normal ones with high sensitivity and specificity.

**Table 2:** Risk score analysis of oesophageal squamous cell carcinoma (ESCC) patients and normal controls.

Score	0–0.764	>0.764	PPV^1^	NPV^2^
Normal subject	51	12		0.81
ESCC	12	51	0.81	
Total	63	63		

1 PPV, positive predictive value; ^2^NPV, negative predictive value.

To further test the specificity of the selected seven miRNAs for ESCC, we also measured these miRNA in sera from patients with two other digestive system tumors including gastric cancer and hepatocarcinoma, and found that only miR-193a-3p was significantly increased in sera from hepatocarcinoma patients as compared with that of normal controls (*P*<0.001), while all the other six miRNAs did not display statistically difference between the patients and normal controls (Figure S2).

### Alteration of the selected miRNAs after operation

**Figure 4:**
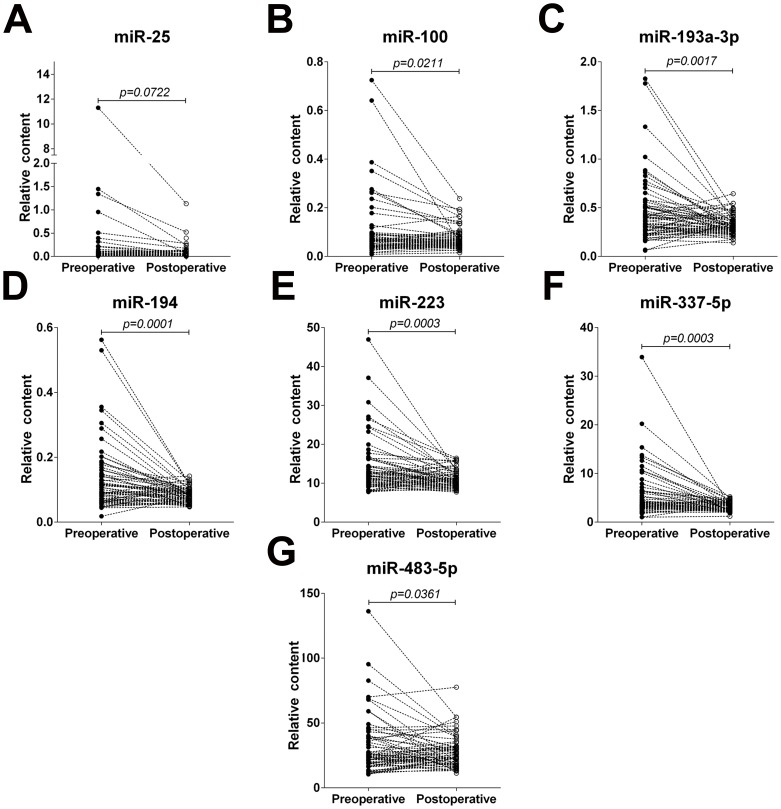
The relative contents of the seven selected serum miRNAs in sera from oesophageal squamous cell carcinoma (ESCC) patients before and after surgery. (A–G) The contents of seven miRNAs including miR-25, miR-100, miR-193-3p, miR-194, miR-223, miR-337-5p and miR-483-5p in paired serum samples of 63 ESCC patients before and 7–10 days after an esophagectomy were assayed by RT-qPCR assay. The relative levels of miRNAs were normalized to let-7-d/g/i and calculated using the 2^−ΔΔCq^ method.

Next, we asked whether the seven selected miRNAs were useful in evaluating the tumour dynamics of ESCC. Therefore, we compared the contents of these miRNAs in paired serum samples of 63 ESCC patients before and 7–10 days after an esophagectomy. As a result, six of the miRNAs, except miR-25, were observed to be significantly reduced after surgical removal of the tumours (at least *P*<0.05), while still higher than normal control (*P*<0.001) (Figure 4A–4G; Table S9; Figure S3).

### Prediction of the ESCC patient survival rate using serum miRNAs

**Figure 5:**
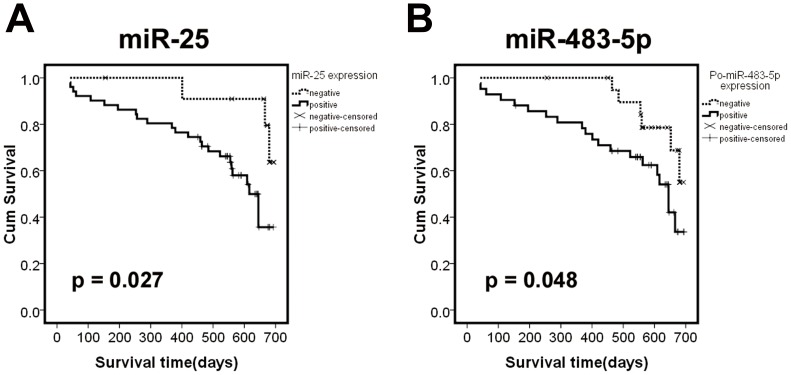
Kaplan–Meier curve estimates the association of miRNAs and the survival of oesophageal squamous cell carcinoma (ESCC) patients. (A)The miR-25 high-level group (greater than the cut-off value; n = 51) compared with the miR-25 low-level group (less than the cut-off value; n = 12). (B) Postoperative serum miR-483-5p high-level group (greater than the cut-off value; n = 48) compared with the miR-483-5p low-level group (less than the cut-off value; n = 15). The OS was defined as the interval between surgery and death or the last follow-up examination. Statistical significance was calculated using the log-rank test.

To explore the association between the serum miRNA levels and the survival rate of ESCC patients, OS curves of ESCC patients were estimated using the Kaplan –Meier method and compared with the log-rank test, using time from cancer onset until death or by censoring at the last follow-up date in the validation set. Of the 63 patients, 58 received the same chemoradiation regimen with paclitaxel (135 mg/m^2^) and platinum (20 mg/m^2^) for sequential chemotherapy in 4 weeks after resection and external irradiation treated by 15 MV-X-ray irradiation in 6 weeks after resection, total dose was 60Gy/25f. Even though the median follow-up time of 19.6 months was relatively short, we could divide the 63 patients into two reasonably sized groups: 27 patients with poor prognosis (dead) and 36 patients with good prognosis (alive without disease). As shown in Figs. 5A–5B, ESCC patients with high levels of miR-25 in their preoperative serum and miR-483-5p in their postoperative serum exhibited lower survival rates than those with low levels (log-rank test: *P* = 0.027 and *P* = 0.048, respectively). High miR-100 levels were also independently associated with an unfavorable outcome (log-rank test: *P* = 0.05). Subsequently, we performed a univariate Cox proportional hazards regression analysis to determine the influence of serum miRNA levels and clinicopathological characteristics on patient survival. We found that regional lymph node metastasis and tumor, node, metastasis stage (TNM stage) were significantly correlated with OS (*P* = 0.011 and *P* = 0.015, respectively) (Table 3). In addition, serum miR-25 and miR-100 levels were associated with survival, though their *P* values were not significant (*P* = 0.076 and *P* = 0.073, respectively). To further explore the impact of miRNA levels in the presence of other prognostic markers, we fitted additional multivariable models for outcome. All factors, including age, sex, TNM stage, lymph node metastasis and histological type, and the miRNA levels were retained in these models irrespective of statistical significance. Multivariable Cox regression analysis indicated that lymph node metastasis status, serum levels of miR-25 and miR-100 were the independent risk factors for OS (*P* = 0.006, hazard ratio (HR)  = 2.98; *P* = 0.046, HR = 3.84; and *P* = 0.024, HR = 4.18, respectively) (Table 3). Collectively, our data suggest that serum levels of miR-25 and miR-100 could potentially serve as prognostic biomarkers for ESCC.

**Table 3:** Univariate and multivariate analyses of parameters associated with overall survival of all oesophageal squamous cell carcinoma (ESCC) patients.

Factors	Univariate analysis				Multivariate analysis			
	HR^1^	95% CI^2^		*P*-value	HR	95% CI		*P-*value
Age (<60/≥60)	0.84	0.39	1.81	0.647	-	-	-	0.295
Sex (male/female)	0.94	0.32	2.73	0.910	-	-	-	0.447
Stage (I, II/III, IV)	2.84	1.23	6.57	0.015	-	-	-	0.476
Lymph node metastasis (absent/present)	2.73	1.26	5.90	0.011	2.98	1.36	6.55	0.006
Differentiation grade (high, middle/low)	1.81	0.83	3.94	0.134	-	-	-	0.659
miR-193a-3p (low/high)	0.89	0.35	2.26	0.810	-	-	-	0.794
miR-194 (low/high)	0.43	0.19	0.98	0.045	-	-	-	0.052
miR-337-5p (low/high)	0.82	0.33	2.03	0.666	-	-	-	0.819
miR-25 (low/high)	3.06	0.89	10.52	0.076	3.84	1.02	14.41	0.046
miR-100 (low/high)	3.00	0.90	9.99	0.073	4.18	1.21	14.50	0.024
miR-223 (low/high)	1.25	0.54	2.90	0.603				0.089
miR-483-5p (low/high)	1.65	0.69	3.92	0.260	-	-	-	0.220
Risk score (low≤0.764/high>0.764)	1.19	0.41	3.49	0.748	-	-	-	0.341

1 HR, hazard ratio; ^2^CI, confidence interval.

## Discussion

In this study, we systematically examined the serum miRNA profile in ESCC using the TaqMan Low Density Array combined with a literature review and individual RT-qPCR validation and found a new panel of seven miRNAs (including miR-25, miR-100, miR-193-3p, miR-194, miR-223, miR-337-5p and miR-483-5p) that could clearly differentiate ESCC patients from normal controls. Because of the similarities among various tumors, such as unlimited proliferation and rapid metastasis, the upregulation of some of these miRNAs is likely to be observed in the sera of patients with other types. Of the seven miRNAs identified in the ESCC patients, several have been reported to be upregulated in serum/plasma from patients with other cancers. For instance, miR-483-5p and miR-193a-3p have been reported to be upregulated in the serum samples of patients with adrenocortical cancer and colorectal cancer, respectively [20]–[21]. miR-194 has been found to be increased in prostate cancer [22]. Increased content of miR-25, on the other hand, has been observed in pancreatic cancer, breast cancer and other cancers [23], [24]. However, the panel of the identified seven miRNAs in our study hasn't been reported to be dysregulated in the serum/plasma of patients with any other cancer. We also examined these miRNAs in patients with two other digestive system tumors to further test their specificity for ESCC. We found that most of the miRNAs didn't exhibit significant increase in sera from patients with hepatocarcinoma and gastric cancer as compared with normal controls. Therefore, the combination of the seven serum miRNAs is more specific than the single miRNA based assay proposed for diagnosis of ESCC. Nevertheless, future studies are necessary to clarify whether the concentration profile for these seven serum miRNAs is capable of discriminating ESCC from other types of tumors.

To date, only three individual miRNAs, namely, miR-21, miR-375 and miR-18a, have been examined in paired preoperative and postoperative serum/plasma samples from ESCC patients who underwent a curative esophagectomy. Of these, miR-21 and miR-18a were found to be significantly higher in ESCC patients than in healthy volunteers and significantly lower in postoperative samples than in preoperative samples [5], [12], [25]. Therefore, the two miRNAs were considered to have the ability to monitor tumour dynamics of ESCC [5], [12], [25]. The sample sizes in these studies were small, with only eight and fourteen ESCC patients studied, respectively [12], [25]. In the current study, we compared a panel of circulating miRNAs in a large cohort of ESCC patients before and after tumor removal. Our results revealed a novel miRNA signature including miR-100, miR-193-3p, miR-194, miR-223, miR-337-5p and miR-483-5p whose levels decreased significantly in the sera of patients after ESCC surgery. It has been reported that some miRNAs are tumour-derived [10]. Our previous study also demonstrated that serum miRNAs were derived not only from circulating blood cells but also from the tissues affected by disease such as tumor tissue [6]. The active secretion or passive release by tumor cells might be one of the major reasons for the differential expression of circulating miRNAs in cancer patients. Although the six miRNAs in the post-operative serum samples didn't revert to the normal lever, their marked reduction after surgical removal of the tumours partly confirm tumour secretion or release of some circulating miRNAs. However, further studies are needed to clarify this issue.

In addition, only a few studies have focused on the role of serum miRNAs in the prognosis of ESCC and four miRNAs, namely, miR-21, miR-31, miR-375 and miR-1246, were found to be associated with overall survival [5], [14], [15]. We also evaluated the potential of miRNAs as prognostic markers for ESCC. Most of patients in our study (92%) received the same treatment modalities. Kaplan-Meier curve analysis revealed that ESCC patients with high levels of miR-25 in their preoperative serum had a significantly shortened OS. Furthermore, we performed univariate and multivariate Cox proportional hazards regression analyses on the OS of the patients and found that the serum levels of miR-25 and miR-100 were the only independent risk factors for OS, suggesting their potential in the predicting the prognosis of ESCC.

Proper normalization is critical for accurate miRNAs quantification. Up to now, several different normalization methods have been applied for measuring circulating miRNAs, including normalizing miRNA concentration to serum volume, or using reference genes including small RNAs, endogenous miRNAs or external non-human miRNAs. Our study and others have clearly demonstrates that small RNAs such as U6, 5S, RNU44 and RNU48 varied greatly in serum and were unstable even after short-term storage [19], [26]. Some endogenous miRNAs like miR-16 and miR-191, which have been commonly used as internal reference for circulating miRNA analysis in cancer patients including ESCC, have been reported to be poor normalizing factors [19], [26]. In addition, spike-in controls are also not an ideal choice, because spike-in controls do not correct for variability in sample collection and therefore cannot improve assay precision [19]. In this study, we used a combination of let-7d, let-7g and let-7i as a reference for the normalization of serum miRNAs. In our previous study, we have systematically evaluated let-7d/g/i and demonstrated it is statistically a superior normalization method as it corrects experimental variations better than existing methods and achieves accurate identification where others do not [19]. Consistent with this previous study, we observed in this study that let-7d/g/i remained constant levels between ESCC and controls. Therefore, let-7d/g/i can be used as an appropriate reference gene for normalization of serum miRNAs, and it can greatly improve sensitivity and reproducibility and successfully guarantee reliable biomarker discovery in our study.

The expression pattern and functional studies of miRNAs in tumour tissue could be helpful in evaluating the clinical application of serum miRNAs. miR-25 has been reported to be upregulated in ESCC tissue compared with the adjacent normal tissue and to function as oncomiRs. The upregulation of miR-25 was significantly correlated with the status of lymph node metastasis and TNM stage [27], [28]. Furthermore, miR-25 was found to promote ESCC cell migration and invasion by suppressing the expression of E-cadherin (CDH1), a very important tumour metastasis suppressor. While miR-100 acts as an anti-oncomiR in ESCC, because miR-100 is found to be downregulated in both ESCC surgical tissue samples and cell lines and involved in invasion and metastasis of ESCC [29]. Moreover, miR-100 can inhibit cell proliferation by suppressing the mTOR oncogene in ESCC cell lines [29]. However, the reason for the inconformity in the expression pattern of miR-100 between serum and tissue samples remains unknown. We suspect that miR-100 could be released from tumour cells into the circulation mainly via an active secretion pattern because many tumours have the remarkable ability to manipulate their stromal environments to their advantage. Further studies are necessary to clarify this issue.

In summary, we have defined a novel distinctive serum miRNA panel for ESCC. Specifically, we have clearly demonstrated that miR-25 and miR-100 can serve as potential biomarkers for predicting the prognosis of ESCC.

## Supporting Information

Figure S1 **The Cq value of let-7d/g/i in serum samples from ESCC patients and normal subjects.** The Cq values of let-7d/g/i in 63 patients with ESCC and 63 normal subjects were assayed by RT-qPCR. The data are presented as means ± SEM deviation.
(TIF)

Figure S2 **The selected seven miRNAs contents in serum samples from patients with other digestive system tumors.** The contents of seven miRNAs including miR-25, miR-100, miR-193-3p, miR-194, miR-223, miR-337-5p and miR-483-5p were measured in sera from gastric cancer (n = 20), hepatocarcinoma (n = 15) patients and normal subjects (n = 20) by RT-qPCR assay. The relative levels of the seven miRNAs were normalized to let-7d/g/i and calculated using the 2^−ΔΔCq^ method. Error bars  = SEM. Asterisks refer to *P*<0.001.
(TIF)

Figure S3 **The comparision of selected seven miRNAs between the post-operation ESCC patients and healthy controls.** The contents of seven miRNAs including miR-25, miR-100, miR-193-3p, miR-194, miR-223, miR-337-5p and miR-483-5p were measured in sera from post-operation ESCC patients (n = 63) and normal subjects (n = 63) by RT-qPCR assay. The relative levels of the seven miRNAs were normalized to let-7d/g/i and calculated using the 2^−ΔΔCq^ method. Error bars  =  SEM. Asterisks refer to *P*<0.001.
(TIF)

Table S1 **The product/catalog numbers of miRNAs for the Applied Biosystems miRNA RT-qPCR assays.**
(DOCX)

Table S2 **Demographic and clinical features of the ESCC patients and normal controls in TaqMan Low Density Assay.**
(DOCX)

Table S3 **Up-regulated miRNAs in ESCC pooled serum sample compared to control sample determined by TaqMan Low Density Assay.**
(DOCX)

Table S4 **The raw Cq value of the seven selected serum miRNA and individual-specific relevant clinical data for all patients and controls of the present study.**
(XLSX)

Table S5 **Significantly increased miRNAs in ESCC serum samples compared to normal controls validated by RT-qPCR.**
(DOCX)

Table S6 **The relative levels of selected seven miRNAs in testing cohort validated by RT-qPCR.**
(DOCX)

Table S7 **Area under the curve and the asymptotic 95% confidence interval of the individual miRNA, the panel of seven-miRNA and CEA for the serum samples in the validation cohort.**
(DOCX)

Table S8 **Area under the curve and the asymptotic 95% confidence interval of the individual miRNA, the panel of seven-miRNA for the serum samples in the testing cohort.**
(DOCX)

Table S9 **The selected seven miRNAs contents in 63 paired pre- and post-operation ESCC serum samples.**
(DOCX)
