# Supplementary figures and images for: Diagnostic and Prognostic Implications of a Serum miRNA Panel in Oesophageal Squamous Cell Carcinoma
Source: PLoS One. 2014 Mar 20;9(3):e92292. doi: 10.1371/journal.pone.0092292 (PMC3961321; doi:10.1371/journal.pone.0092292)

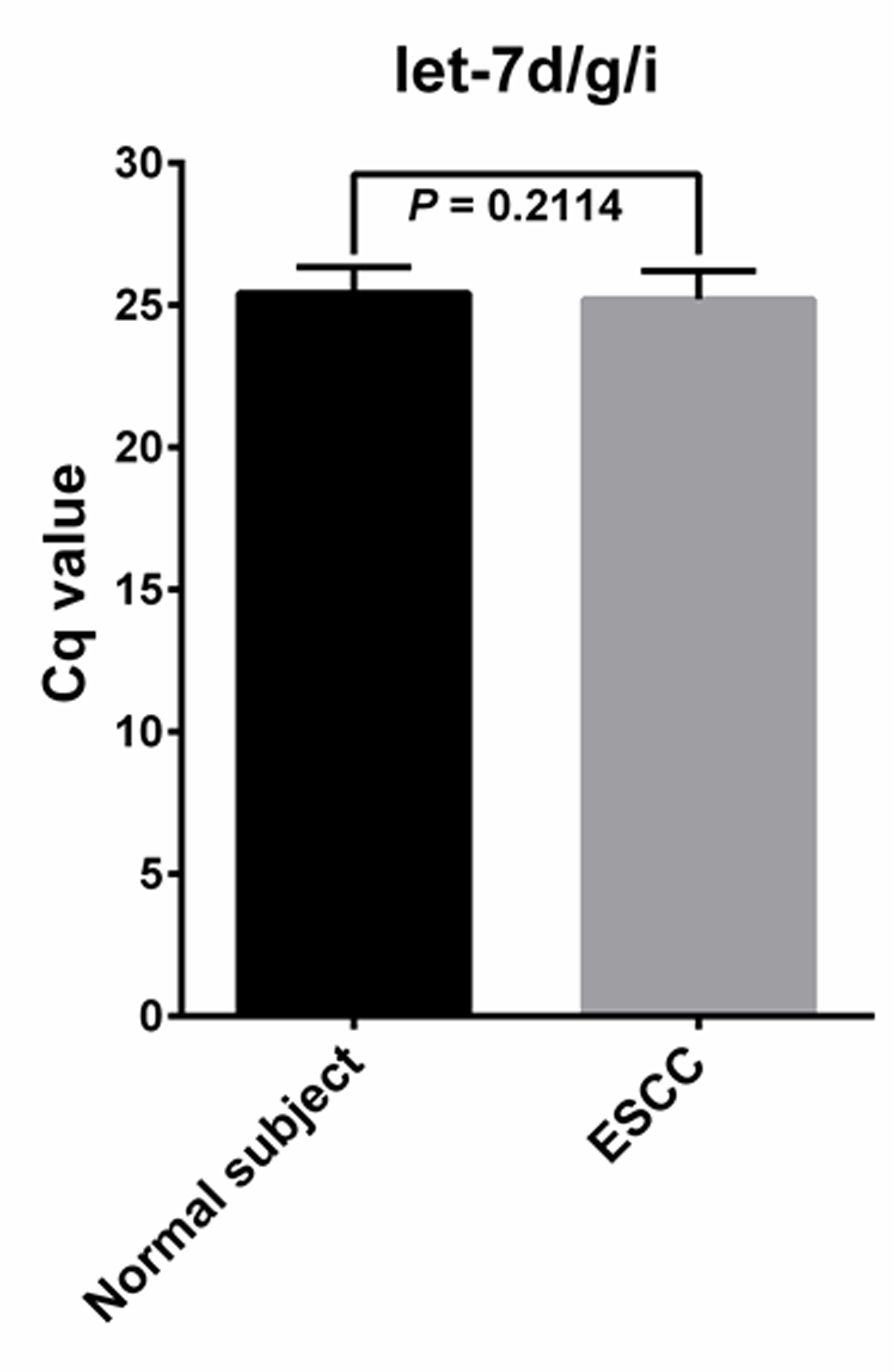

Supplement: Figure S1 — The Cq value of let-7d/g/i in serum samples from ESCC patients and normal subjects. The Cq values of let-7d/g/i in 63 patients with ESCC and 63 normal subjects were assayed by RT-qPCR. The data are presented as means ± SEM deviation. (TIF) [file pone.0092292.s001.tif]

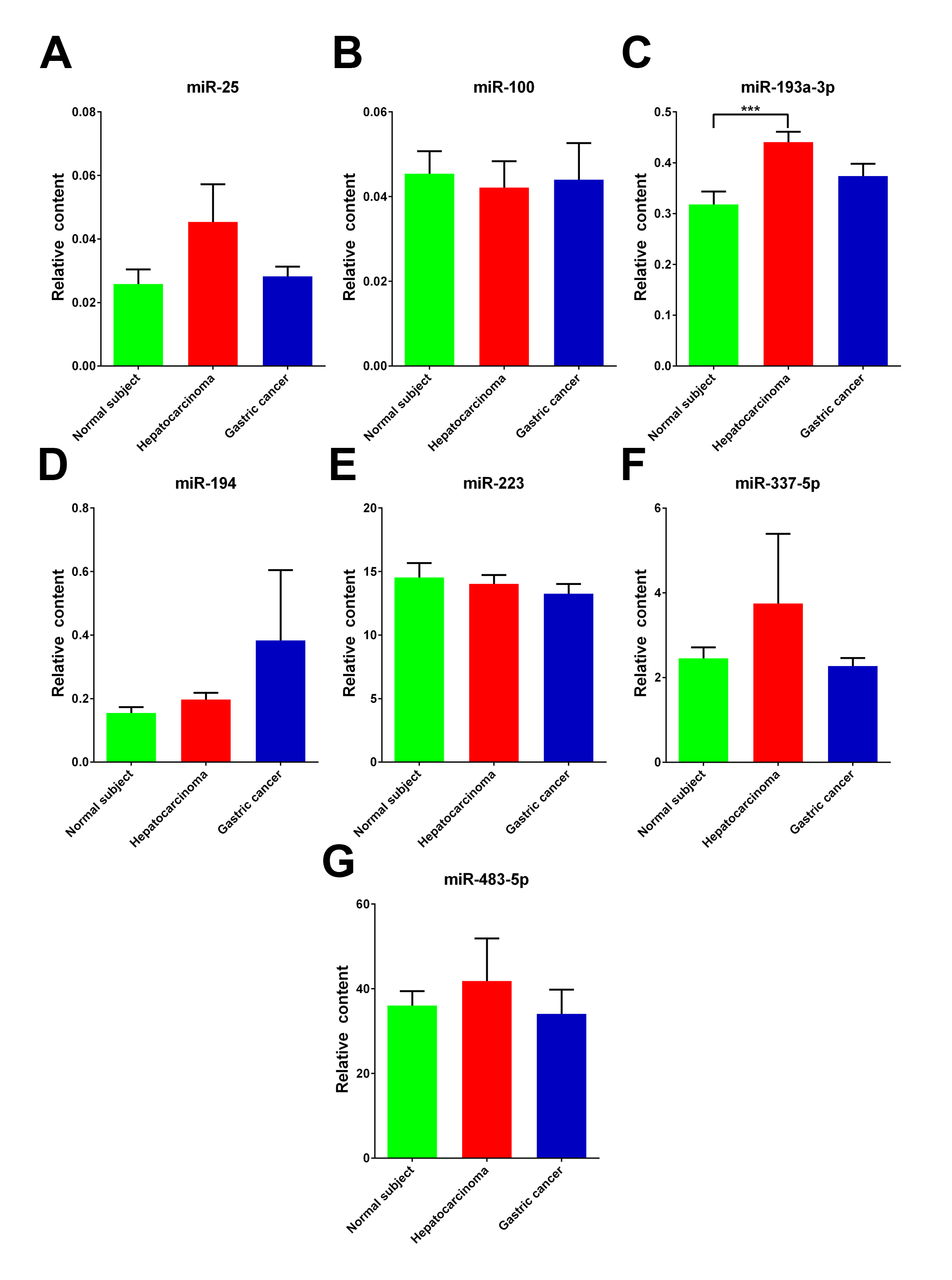

Supplement: Figure S2 — The selected seven miRNAs contents in serum samples from patients with other digestive system tumors. The contents of seven miRNAs including miR-25, miR-100, miR-193-3p, miR-194, miR-223, miR-337-5p and miR-483-5p were measured in sera from gastric cancer (n = 20), hepatocarcinoma (n = 15) patients and normal subjects (n = 20) by RT-qPCR assay. The relative levels of the seven miRNAs were normalized to let-7d/g/i and calculated using the 2−ΔΔCq method. Error bars = SEM. Asterisks refer to P<0.001. (TIF) [file pone.0092292.s002.tif]

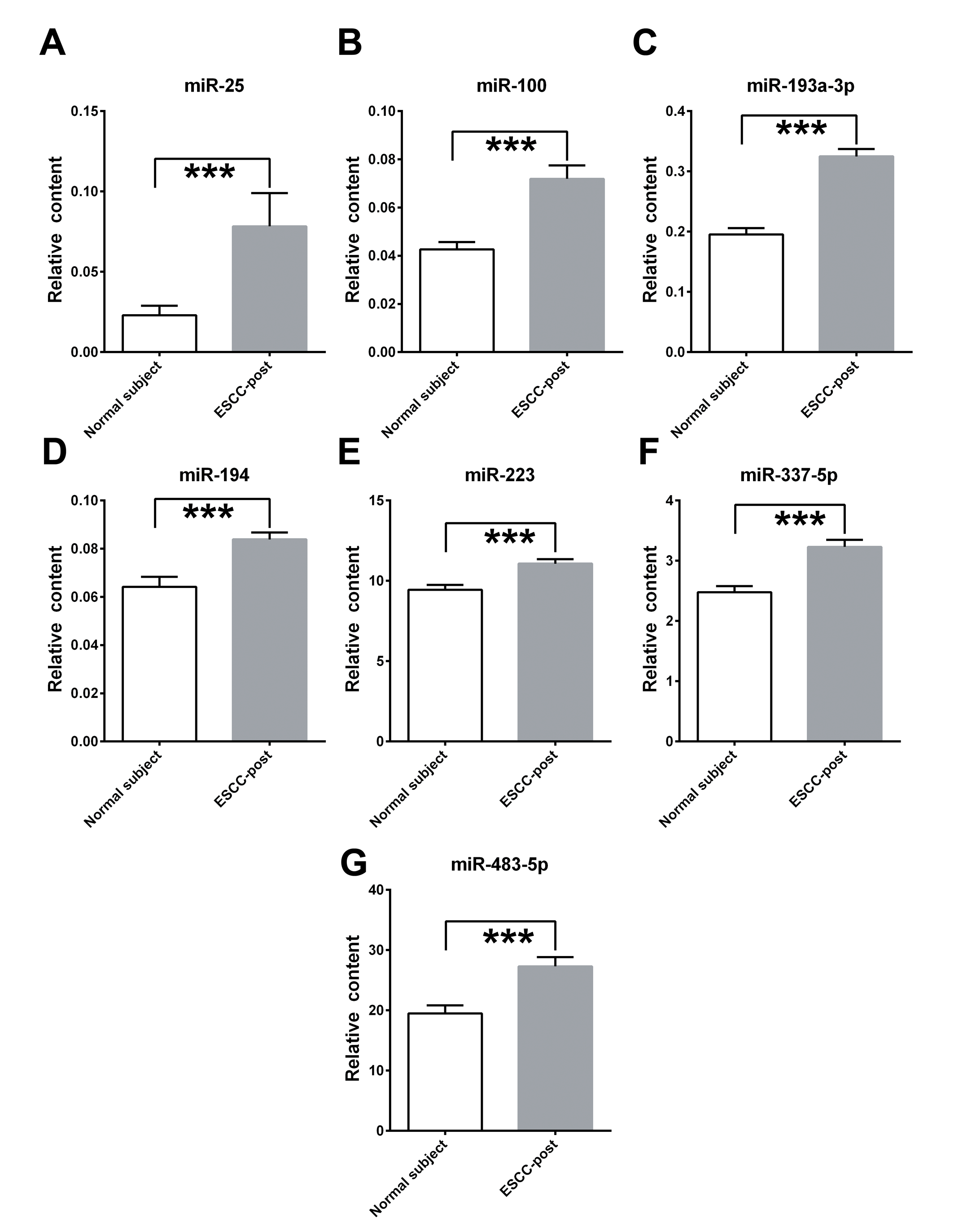

Supplement: Figure S3 — The comparision of selected seven miRNAs between the post-operation ESCC patients and healthy controls. The contents of seven miRNAs including miR-25, miR-100, miR-193-3p, miR-194, miR-223, miR-337-5p and miR-483-5p were measured in sera from post-operation ESCC patients (n = 63) and normal subjects (n = 63) by RT-qPCR assay. The relative levels of the seven miRNAs were normalized to let-7d/g/i and calculated using the 2−ΔΔCq method. Error bars = SEM. Asterisks refer to P<0.001. (TIF) [file pone.0092292.s003.tif]
