# Supplementary material for: Diagnostic and Prognostic Implications of a Serum miRNA Panel in Oesophageal Squamous Cell Carcinoma
Source: PLoS One. 2014 Mar 20;9(3):e92292. doi: 10.1371/journal.pone.0092292 (PMC3961321; doi:10.1371/journal.pone.0092292)
Supplement: Table S1 — The product/catalog numbers of miRNAs for the Applied Biosystems miRNA RT-qPCR assays. (DOCX) [file pone.0092292.s004.docx]

**Table S1** The product/catalog numbers of miRNAs for the Applied Biosystems

miRNA RT-qPCR assays.

| miRNA | Cat. number | Product number |
| --- | --- | --- |
| miR-483-5p | Cat. # 4427975 | 002338 |
| miR-193a-3p | Cat. # 4427975 | 002250 |
| miR-25 | Cat. # 4427975 | 002442 |
| miR-337-5p | Cat. # 4427975 | 002156 |
| miR-194 | Cat. # 4427975 | 000493 |
| miR-223 | Cat. # 4427975 | 002295 |
| miR-100 | Cat. # 4427975 | 000437 |
| miR-7 | Cat. # 4427975 | 000268 |
| miR-198 | Cat. # 4427975 | 002273 |
| miR-216a | Cat. # 4427975 | 002220 |
| miR-1247 | Cat. # 4427975 | 002893 |
| let-7d | Cat. # 4427975 | 002283 |
| let-7g | Cat. # 4427975 | 002282 |
