# Supplementary material for: Diagnostic and Prognostic Implications of a Serum miRNA Panel in Oesophageal Squamous Cell Carcinoma
Source: PLoS One. 2014 Mar 20;9(3):e92292. doi: 10.1371/journal.pone.0092292 (PMC3961321; doi:10.1371/journal.pone.0092292)
Supplement: Table S2 — Demographic and clinical features of the ESCC patients and normal controls in TaqMan Low Density Assay. (DOCX) [file pone.0092292.s005.docx]

**Table S2** Demographic and clinical features of the ESCC patients and normal controls in TaqMan Low Density Assay.^1^

|  | ESCC (n = 28) | | Controls (n = 28) | | *P-*value |
| --- | --- | --- | --- | --- | --- |
| Variables | No. | % | No. | % |  |
| **Age (years)** | 63.1±8.8 | | 62.4±3.2 | | *P* = 0.5668^2^ |
| **Sex** |  |  |  |  | *P* = 0.7674^3^ |
| Male | 21 | 75 | 19 | 21 |  |
| Female | 7 | 25 | 9 | 7 |  |
| **Metastasis** |  |  |  |  |  |
| Yes | 25 | 89 |  |  |  |
| No | 3 | 11 |  |  |  |
| **Differentiation grade** |  |  |  |  |  |
| High | 3 | 10.7 |  |  |  |
| Middle | 8 | 28.6 |  |  |  |
| Low | 17 | 60.7 |  |  |  |
| **TNM stage** |  |  |  |  |  |
| I | 5 | 17.9 |  |  |  |
| II | 9 | 32.1 |  |  |  |
| III | 10 | 35.8 |  |  |  |
| IV | 2 | 7.1 |  |  |  |
| Unknown | 2 | 7.1 |  | 2 |  |

^1^Data are mean±SD. *P*^2^: student-*t* test; *P*^3^: two-sided χ^2^ test.
