# Supplementary material for: Diagnostic and Prognostic Implications of a Serum miRNA Panel in Oesophageal Squamous Cell Carcinoma
Source: PLoS One. 2014 Mar 20;9(3):e92292. doi: 10.1371/journal.pone.0092292 (PMC3961321; doi:10.1371/journal.pone.0092292)
Supplement: Table S3 — Up-regulated miRNAs in ESCC pooled serum sample compared to control sample determined by TaqMan Low Density Assay. (DOCX) [file pone.0092292.s006.docx]

**Table S3** Up-regulated miRNAs in ESCC pooled serum sample compared to control sample determined by TaqMan Low Density Assay.

| miRNA | ESCC |  | Normal controls | | △△Cq | Fold  change |
| --- | --- | --- | --- | --- | --- | --- |
|  | Cq | △Cq | Cq | △Cq |  |  |
| hsa-miR-198 | 19.15052 | 3.4587002 | Undetermined | 23.268715 | -19.81 | 919197.2 |
| hsa-miR-337-5p | 19.970259 | 4.2784386 | Undetermined | 23.268715 | -18.9903 | 520766.2 |
| hsa-miR-216a | 20.810268 | 5.1184483 | Undetermined | 23.268715 | -18.1503 | 290920.8 |
| hsa-miR-193a-3p | 22.983116 | 7.291296 | 31.957802 | 15.226517 | -7.93522 | 244.7595 |
| hsa-miR-194 | 23.850977 | 8.159157 | 30.012842 | 13.281558 | -5.1224 | 34.83344 |
| hsa-miR-1247 | 24.948334 | 10.156294 | Undetermined | 23.296312 | -13.14 | 9026.919 |
| hsa-miR-218-1* | 26.890245 | 12.098206 | Undetermined | 23.296312 | -11.1981 | 2349.448 |
| hsa-miR-593 | 27.007849 | 12.215809 | Undetermined | 23.296312 | -11.0805 | 2165.528 |
| hsa-miR-302b | 29.743593 | 14.051773 | Undetermined | 23.268715 | -9.21694 | 595.0809 |
| hsa-miR-367 | 29.864431 | 14.172611 | Undetermined | 23.268715 | -9.0961 | 547.2681 |
| hsa-miR-130a* | 29.048683 | 14.256642 | Undetermined | 23.296312 | -9.03967 | 526.2739 |
| hsa-miR-488 | 30.028929 | 15.236889 | Undetermined | 23.296312 | -8.05942 | 266.7645 |
| hsa-miR-422a | 30.96281 | 15.270991 | Undetermined | 23.268715 | -7.99772 | 255.5965 |
| hsa-miR-548a | 31.950626 | 16.258806 | Undetermined | 23.268715 | -7.00991 | 128.8822 |
| hsa-miR-767-5p | 31.728994 | 16.936954 | Undetermined | 23.296312 | -6.35936 | 82.10271 |
| hsa-miR-23a* | 31.952776 | 17.160736 | Undetermined | 23.296312 | -6.13558 | 70.306 |
| hsa-miR-576-3p | 32.931522 | 17.239702 | Undetermined | 23.268715 | -6.02901 | 65.30009 |
| hsa-miR-125b-1* | 33.990234 | 19.198195 | Undetermined | 23.296312 | -4.09812 | 17.12601 |
| hsa-miR-545 | 34.984947 | 19.293127 | Undetermined | 23.268715 | -3.97559 | 15.73154 |
| hsa-miR-142-5p | 35.013947 | 19.322126 | Undetermined | 23.268715 | -3.94659 | 15.41848 |
| hsa-miR-548I | 34.852757 | 20.060717 | Undetermined | 23.296312 | -3.2356 | 9.419138 |
| hsa-miR-548c | 35.92828 | 20.23646 | Undetermined | 23.268715 | -3.03226 | 8.180874 |
| hsa-miR-520e | 36.048824 | 20.357004 | Undetermined | 23.268715 | -2.91171 | 7.525101 |
| hsa-miR-1256 | 35.89231 | 21.100271 | Undetermined | 23.296312 | -2.19604 | 4.582202 |
| hsa-miR-449 | 37.120476 | 21.428656 | Undetermined | 23.268715 | -1.84006 | 3.580247 |
| hsa-miR-148b* | 33.956215 | 19.164175 | 36.96684 | 20.263151 | -1.09898 | 2.142026 |
