# Supplementary material for: Diagnostic and Prognostic Implications of a Serum miRNA Panel in Oesophageal Squamous Cell Carcinoma
Source: PLoS One. 2014 Mar 20;9(3):e92292. doi: 10.1371/journal.pone.0092292 (PMC3961321; doi:10.1371/journal.pone.0092292)
Supplement: Table S5 — Significantly increased miRNAs in ESCC serum samples compared to normal controls validated by RT-qPCR. (DOCX) [file pone.0092292.s008.docx]

**Table S5** Significantly increased miRNAs in ESCC serum samples compared to normal controls validated by RT-qPCR.^1^

| miRNA | ESCC (n = 63) | Controls (n = 63) | *P-*value |
| --- | --- | --- | --- |
| miR-7 | 0.66±0.03 | 0.80±0.05 | 0.0399 |
| miR-25 | 0.40±0.20 | 0.02±0.01 | < 0.0001 |
| miR-100 | 0.10±0.02 | 0.04±0.00 | < 0.0001 |
| miR-193a-3p | 0.46±0.04 | 0.20±0.01 | < 0.0001 |
| miR-194 | 0.13±0.01 | 0.06±0.00 | < 0.0001 |
| miR-198 | 0.21±0.03 | 0.13±0.02 | 0.0934 |
| miR-216a | 0.17±0.02 | 0.15±0.01 | 0.4283 |
| miR-223 | 14.22±0.91 | 9.45±0.30 | < 0.0001 |
| miR-337-5p | 5.69±0.66 | 2.48±0.10 | < 0.0001 |
| miR-483-5p | 32.54±2.83 | 19.51±1.34 | < 0.0001 |
| miR-1247 | 11.86±1.81 | 3.70±0.80 | 0.0002 |

^1^The relative contents of miRNAs are presented as mean±SEM.
