# Supplementary material for: Diagnostic and Prognostic Implications of a Serum miRNA Panel in Oesophageal Squamous Cell Carcinoma
Source: PLoS One. 2014 Mar 20;9(3):e92292. doi: 10.1371/journal.pone.0092292 (PMC3961321; doi:10.1371/journal.pone.0092292)
Supplement: Table S6 — The relative levels of selected seven miRNAs in testing cohort validated by RT-qPCR. (DOCX) [file pone.0092292.s009.docx]

**Table S6** The relative levels of selected seven miRNAs in testing cohort validated by RT-qPCR.^1^

| miRNA | ESCC (n = 20) | Controls (n = 20) | *P-*value |
| --- | --- | --- | --- |
| miR-25 | 0.09±0.01 | 0.03±0.01 | <0.0001 |
| miR-100 | 0.09±0.01 | 0.04±0.01 | <0.0001 |
| miR-193a | 0.70±0.02 | 0.26±0.02 | <0.0001 |
| miR-194 | 0.22±0.01 | 0.11±0.01 | <0.0001 |
| miR-223 | 20.63±1.30 | 11.00±1.39 | <0.0001 |
| miR-337-5p | 3.46±0.356 | 2.08±0.18 | 0.0018 |
| miR-483-5p | 29.956±2.41 | 22.01±2.73 | 0.0103 |

^1^The relative contents of miRNAs are presented as mean±SEM.
