# Supplementary material for: Diagnostic and Prognostic Implications of a Serum miRNA Panel in Oesophageal Squamous Cell Carcinoma
Source: PLoS One. 2014 Mar 20;9(3):e92292. doi: 10.1371/journal.pone.0092292 (PMC3961321; doi:10.1371/journal.pone.0092292)
Supplement: Table S7 — Area under the curve and the asymptotic 95% confidence interval of the individual miRNA, the panel of seven-miRNA and CEA for the serum samples in the validation cohort. (DOCX) [file pone.0092292.s010.docx]

**Table S7** Area under the curve and the asymptotic 95% confidence interval of the individual miRNA, the panel of seven-miRNA and CEA for the serum samples in the validation cohort.

| miRNA | Area | Std. Error | Asymptotic Sig. | Asymptotic 95%  Confidence Interval | |
| --- | --- | --- | --- | --- | --- |
|  |  |  |  | Lower Bound | Upper Bound |
| miR-193-3p | 0.85 | 0.03 | 0.000 | 0.78 | 0.92 |
| miR-194 | 0.81 | 0.04 | 0.000 | 0.73 | 0.89 |
| miR-337-5p | 0.85 | 0.04 | 0.000 | 0.78 | 0.92 |
| miR-25 | 0.78 | 0.04 | 0.000 | 0.70 | 0.86 |
| miR-100 | 0.75 | 0.04 | 0.000 | 0.66 | 0.83 |
| miR-223 | 0.77 | 0.04 | 0.000 | 0.69 | 0.85 |
| miR-483-5p | 0.74 | 0.05 | 0.000 | 0.65 | 0.83 |
| Panel | 0.83 | 0.04 | 0.000 | 0.75 | 0.90 |
| CEA | 0.60 | 0.06 | 0.084 | 0.47 | 0.72 |
