# Supplementary material for: Diagnostic and Prognostic Implications of a Serum miRNA Panel in Oesophageal Squamous Cell Carcinoma
Source: PLoS One. 2014 Mar 20;9(3):e92292. doi: 10.1371/journal.pone.0092292 (PMC3961321; doi:10.1371/journal.pone.0092292)
Supplement: Table S8 — Area under the curve and the asymptotic 95% confidence interval of the individual miRNA, the panel of seven-miRNA for the serum samples in the testing cohort. (DOCX) [file pone.0092292.s011.docx]

**Table S8** Area under the curve and the asymptotic 95% confidence interval of the individual miRNA, the panel of seven-miRNA for the serum samples in the testing cohort.

| miRNA | Area | Std. Error | Asymptotic Sig. | Asymptotic 95%  Confidence Interval | |
| --- | --- | --- | --- | --- | --- |
|  |  |  |  | Lower Bound | Upper Bound |
| miR-25 | 0.94 | 0.05 | <0.001 | 0.85 | 1.03 |
| miR-100 | 0.88 | 0.06 | <0.001 | 0.76 | 1.01 |
| miR-193a | 1.00 | 0.00 | <0.001 | 0.99 | 1.01 |
| miR-194 | 0.92 | 0.05 | <0.001 | 0.81 | 1.01 |
| miR-223 | 0.90 | 0.06 | <0.001 | 0.77 | 1.02 |
| miR-337-5p | 0.78 | 0.08 | 0.002 | 0.63 | 0.94 |
| miR-483-5p | 0.74 | 0.08 | 0.011 | 0.57 | 0.90 |
| Panel | 0.93 | 0.04 | <0.001 | 0.86 | 1.01 |
