# Supplementary material for: Diagnostic and Prognostic Implications of a Serum miRNA Panel in Oesophageal Squamous Cell Carcinoma
Source: PLoS One. 2014 Mar 20;9(3):e92292. doi: 10.1371/journal.pone.0092292 (PMC3961321; doi:10.1371/journal.pone.0092292)
Supplement: Table S9 — The selected seven miRNAs contents in 63 paired pre- and post-operation ESCC serum samples. (DOCX) [file pone.0092292.s012.docx]

**Table S9** The selected seven miRNAs contents in 63 paired pre- and post-operation ESCC serum samples.^1^

| miRNA | Pre-operation | Post-operation | *P-*value |
| --- | --- | --- | --- |
| miR-25 | 0.40±0.20 | 0.08±0.02 | 0.0722 |
| miR-100 | 0.10±0.02 | 0.07±0.01 | 0.0211 |
| miR-193a-3p | 0.46±0.04 | 0.32±0.01 | 0.0017 |
| miR-194 | 0.13±0.01 | 0.08±0.00 | 0.0001 |
| miR-223 | 14.22±0.91 | 11.07±0.27 | 0.0003 |
| miR-337-5p | 5.69±0.66 | 3.23±0.12 | 0.0003 |
| miR-483-5p | 32.54±2.83 | 27.32±1.54 | 0.0361 |

^1^The relative contents of miRNAs are presented as mean±SEM. *P*1*-*value: Post-operation *vs*. Pre-operation. *P*2*-*value: Post-operation *vs*. Normal controls.
